# A qualitative study on the ethics of transforming care: examining the development and implementation of Canada’s first mental health strategy

**DOI:** 10.1186/s13012-015-0297-y

**Published:** 2015-08-19

**Authors:** Melissa M. Park, Raphael Lencucha, Cheryl Mattingly, Hiba Zafran, Laurence J. Kirmayer

**Affiliations:** 1 School of Physical and Occupational Therapy, Faculty of Medicine, McGill University, 3600 Promenade Sir William Osler, Montreal, QC H3G 1Y5 Canada; 2 Participatory Research at McGill, McGill University, Montreal, QC H3S 1Z1 Canada; 3 Lady Davis Institute for Medical Research, Jewish General Hospital, Montreal, QC H3T 1E2 Canada; 4 Department of Anthropology and the Division of Occupational Science and Therapy, University of Southern California, 3620 S. Vermont Avenue, Los Angeles, CA USA; 5 Dale T. Mortensen Senior Research Fellow at the Institute of Advanced Studies, Aarhus University, Aarhus, Denmark; 6 Division of Social and Transcultural Psychiatry, Department of Psychiatry, McGill University, 1033 Pine Ave, Montreal, QC H3A 1A1 Canada

**Keywords:** Recovery, Policy, Practice guidelines, Everyday ethics, Ethnography, Participatory research

## Abstract

**Background:**

The Mental Health Commission of Canada worked collaboratively with stakeholders to create a new framework for a federal mental health strategy, which is now mandated for implementation by 2017. The proposed strategies have been written into provincial health plans, hospital accreditation standards, and the annual objectives of psychiatric departments and community organizations. This project will explore the decision-making process among those who contributed to Canada’s first federal mental health policy and those implementing this policy in the clinical setting. Despite the centrality of ethical reasoning to the successful uptake of the recent national guidelines for recovery-oriented care, to date, there are no studies focused exclusively on the ethical tensions that emerged and continue to emerge during the creation and implementation of the new standards for recovery-oriented practice.

**Methods/design:**

This two-year Canadian Institute of Health Research Catalyst Grant in Ethics (2015–2017) consists of three components. C-I, a retrospective, qualitative study consisting of document analysis and interviews with key policy-makers of the ethical tensions that arose during the development of Canada’s Mental Health Strategy will be conducted in parallel to C-II, a theory-based, focused ethnography of how mental health practitioners in a psychiatric setting reason about and act upon new standards in everyday practice. Case-based scenarios of ethical tensions will be developed from C-I/II and fed-forward to C-III: participatory forums with policy-makers, mental health practitioners, and other stakeholders in recovery-oriented services to collectively identify and prioritize key ethical concerns and generate action steps to close the gap between the policy-making process and its implementation at the local level.

**Discussion:**

Policy-makers and clinicians make important everyday decisions that effect the creation and implementation of new practice standards. Particularly, there is a need to understand how ethical dilemmas that arise during this decision-making process and the reasoning and resources they use to resolve these tensions impact on the implementation process. This catalyst grant in ethics will (1) introduce a novel line of inquiry focusing on the ethical tensions that arose in the development of Canada’s first mental health strategy, while (2) intensifying our focus on the ethical aspects of moving policy into action.

## Background

The Canadian federal government published its first National Mental Health Strategy [1] based on the work of the Mental Health Commission of Canada’s (MHCC) vision of recovery-oriented services, *Toward Recovery and Well-Being: A Framework for a Mental Health Strategy of Canada* [2]. The orientation to wellness in recovery models stands in contrast to biomedical models that stress the eradication and/or control of the disease. Consequently, the recovery model is a radical or discontinuous innovation that is unlikely to diffuse rapidly or easily in mental health treatment organizations [3]. The inherent conflict between the values, basic concepts, knowledge base, working practices and goals of recovery, and traditional approaches will require more than acquiring a new language or set of skills [4]: “Experience in other countries and here at home tells us that it will take a *sustained action on many fronts to truly shift culture and practice* in the mental health system towards recovery and well being [italics added]” ([1]:36). In essence, transformation of mental health care requires a re-examination of the values that constitute, and who ultimately decides, what *a good life* is for persons with severe mental illness. Such ethical decisions are critical in increasingly heterogeneous local contexts with divergent and, often conflicting, values [5].

As a broad federal policy initiative, the MHCC’s Strategy intentionally lacks precise directives for implementation, allowing for adaptation to the needs of particular individuals and capacity of particular local contexts. This openness provides an opportunity to establish practice that is rooted in a local context and culture [6]. It also reflects the unique and extensive consultation process with stakeholders (i.e., persons with mental illness, family members, organizations, and the general public) taken by the MHCC to shape these initiatives e.g., see [2]. This process represents, if implicitly, a relational ethics perspective being promoted in psychiatry. As the term suggests, relational ethics “situates ethical practice in relationships” ([7]:843) where judgments of the good can be made together [8]. Yet, this openness also reflects the additional task confronting policy-makers of reconciling thousands of different perspectives from first-person accounts of experiences of mental illness with the requirements of a federal mental health policy for the best good of the majority.

The core elements of a relational ethics approach, such as engaged interaction, mutual respect, embodied knowledge, and interdependence, heighten the uncertainty and vulnerability of individuals [7]. This vulnerability is apparent in the MHCC strategy, which used engaged consultation: “The stories we have heard from people living with mental health problems and illnesses, their families, and the many dedicated people who work with them across the country [that] have moved us, have angered us, and have inspired us” [1]. Thus, the openness of the broad federal initiative represents the challenge of how to deliberate about the “best good” for others from a values-based perspective, while also and essentially, passing this relational ethics challenge to mental health practitioners.

More recently, Canada’s Mental Health Strategy was operationalized within new accreditation standards [9], which will require health professionals to align their practice with the recovery-oriented model of care in the next several years. This Catalyst in Ethics project is nested within and provides a critical focus to our CIHR Strategy for Patient-Oriented Research and Rx&D Health Research Foundation Partnerships for Health System Improvement (PHSI) study: *Transforming mental health services: A participatory mixed methods study to promote and evaluate the implementation of recovery-oriented care* (#293636) [10]. Although we understood how recovery-oriented policy would create a radical shift in clinical orientation by emphasizing wellness over symptom management or cure, and responsibility of persons with mental illness for their own care over professional expertise [4], we did not anticipate the extent to which the implementation of recovery-oriented approaches would create ethical tensions for practitioners.

We use the term *ethical tension* to include the moral uncertainty, distress, or dilemmas experienced by individuals [11] that impede the translation of policy into practice. In clinical practice, ethical tensions reposition practitioners as everyday ethicists. *Everyday ethics* is “…the core of ethical behavior between staff and patients that may reside in the seeming minutiae of small social exchanges” ([12]:173), which are heightened during moments of uncertainty [13, 14]. By *everyday ethicists*, we refer to the ways in which policy-makers or practitioners deliberate about the good [15] when facing continued uncertainty about how to deliver the best care to persons with mental illness [16]. Despite the centrality of everyday ethics to the successful uptake of the recent guidelines for recovery-oriented care, to date, there are no studies focused exclusively on the ethical tensions that impede the translation of new mental health policy into practice. Thus, we (1) introduce a novel line of inquiry focusing on the ethical tensions that arose in the development of, and which may remain in, Canada’s first mental health strategy, while (2) intensifying our focus on the ethical aspects of moving policy into action.

Tension already exists in client-centered mental health care between the bioethical principles of beneficence and autonomy [17], such as how assumptions about care may guide practitioners to foster dependency [18]. The core premise of recovery—to empower persons with mental illness and their families—accentuates this tension by overturning previous conceptions of the relationship between mental health systems, practitioners, persons with mental illness, and family members. The need for greater involvement of persons with lived experience of mental illness in the implementation and ownership over their own recovery processes [19] displaces traditional roles by creating a *partnership of mutuality* [4]. However, the concept of a partnership of mutuality increases ethical tensions: for example, when practitioners who might frame the bioethical value of beneficence as protecting individuals from suffering exert authority and diminish the possibility of mutual collaboration over treatment [20]. This raises the question: How do practitioners face such ethical tensions and make decisions about the best course of action to take when faced with multiple and often competing values embedded in their own practice frameworks and guidelines?

Based on observations of this clinical reasoning from ethnographic studies, we know that practitioners rely on both technical and practical rationality [21]. Practitioners use technical rationality to make decisions about best interventions for a disease or disability process based on best evidence and practical rationality to individualize care. Practical rationality, an Aristotelian notion, draws on accumulated experiences with others over time. This experiential knowledge plays a critical role in how practitioners are able to interpret or understand others, in addition to deciding what might be the best intervention to motivate a particular person to engage in and manage their own care [22]. Thus, how practitioners use this experiential knowledge to make decisions about the “best good” is central to understanding the relationship between ethical tensions and how they are resolved during the implementation of recovery-oriented policy. Yet, experiential knowledge is often overlooked and underutilized in knowledge translation efforts to inform health policy [23] and unacknowledged or even considered illegitimate in clinical practice [15, 24].

## Project design: ethical framework

Our focus on everyday ethics directs us to neo-Aristotelian virtue ethics, in which “all human beings, whatever their culture, participate (or try to) in the planning and managing of their lives, asking and answering questions about how one should live and act” ([25]:28). Neo-Aristotelian virtue ethics concerns itself with questions about the “good” as opposed to the “right,” where the good represents the best approach in a particular context, as opposed to means-end utilitarianism, where the right represents the best approach based on general principles or the best available evidence.

On the ground, however, healthcare practitioners toggle back and forth between these different approaches to ethical issues that mirror an ongoing debate in the bioethics community between principlism and narrative ethics [26]. Principlism has dominated bioethics for the past century and fosters a deductive approach to ethical decision-making. Principlists apply predetermined and intellectually reasoned principles (e.g., autonomy, beneficence, and justice) to healthcare, which may require adjustment within different situations. A narrative ethics approach, in contrast, fosters an inductive and interpretive approach to describing and understanding decision-making from the perspective of particular persons in particular situations [27]. Thus, narrativists situate the storyteller at the forefront of the reasoning process with the interpretive intention to “…capture what it is about our lives that matters most to us” ([26]:68).

Since we are most interested in how individuals reason about the best course of action to take in their everyday decisions, we have chosen an ethics framework that is a first-person phenomenological virtue ethics rooted in neo-Aristotelian traditions [15, 28, 29]. As an ethics framework, this analytic approach is grounded in phenomenology and narrative theories [15, 30, 31], which foreground what really matters to ordinary persons during times of uncertainty or irruption [32], such as with the uncertainty created for practitioners, persons with mental illness, and family members with the introduction of recovery-oriented policy on recovery that is changing the culture of mental health practice itself. Policy, accreditation standards and practice guidelines are fundamentally structural in nature and represent norms or social discourses. Thus, our focus on the experiential uncertainty facing particular persons provides a unique and essential window onto the everyday ethical reasoning affecting the uptake and actual use of new policy and models of practice. Developed over a decade and a half of longitudinal ethnographies of health care encounters [33, 34], a narrative-phenomenological framework has three specific levels of analysis [31] best suited to capture the experiences of particular persons around shared events and how persons draw on (or not) social discourses in their reasoning as “judgment rather than an application of general rules to a particular case” ([15]:289). These three analytic levels are:

1. *Person-centered*. Attention is paid to the particularities of actions, persons, and places, including the esthetic qualities of experience [35, 36]. In other words, our analytic framework is directed towards heightened, often shared, experiences, and the meanings individuals attribute to these experiences.
2. *Event-focused*. Events are situations that are subjectively experienced as significant and which, through analysis, can make visible what is at stake for those persons involved [37]. Events, particularly those that deviate from the expected, often signal transformative moments and reveal what matters or the values that guide actions during uncertainty [32].
3. *Discursive practices*. Discourses are defined, from a critical epistemological perspective, as the larger structural forces that guide, often unconsciously, one’s actions [38–40]: the “ensemble of ideas, concepts, and categories through which meaning is given to social and physical phenomena, and which is produced and reproduced through an identifiable set of practices” ([41]:67). We define discursive practices as discourses, which have a narrative structure or form and are (re)produced by individuals through everyday talk or action (see also [5]). For example, individuals enact genres of healing (e.g., machine repair, detective story, battle or transformative journey) in biomedicine to guide their approach to illness [31] or the quest genre drawn from popular culture to guide their actions during illness experiences [42]. There may also be a structure and genre of recovery narratives, much like the narrative of Alcoholics Anonymous [43]. It is also important to note that practices are things people do or reproduce through action, which they may or may not be fully aware of from a first-person perspective. Hence, in order to provide a third-person perspective on discursive practices, we will supplement interviews with participant observations to address precisely this problem.

These three levels of analysis are key to understanding the complex interplay between sociocultural structures (discourses), habitual action (practices), and improvisational action (agency). A first-person perspective provides a unique and essential lens to examine the ethical tensions related to the development or implementation of policy and how persons reflect on, reason about, or enact (or not).

## Project design: methodology

The overarching aim of this project is to examine the ethics of mental health policy development and implementation. Using a mixed methods qualitative design, we seek to understand the ethical tensions that arise during policy-making and its implementation that accentuate the gap between policy and everyday practice. Our research questions are bi-directional and sequential and correspond to three components. Component-I and II (C-I and II) will be conducted in parallel and are situated within an interpretivist paradigm, which recognizes the subjectivity involved in knowing and, particularly, in what we can understand of the experiences of another individual. Emergent findings will be fed-forward to Component-III (C-III), which is situated within a transformative paradigm (Table 1).

**Table 1:**
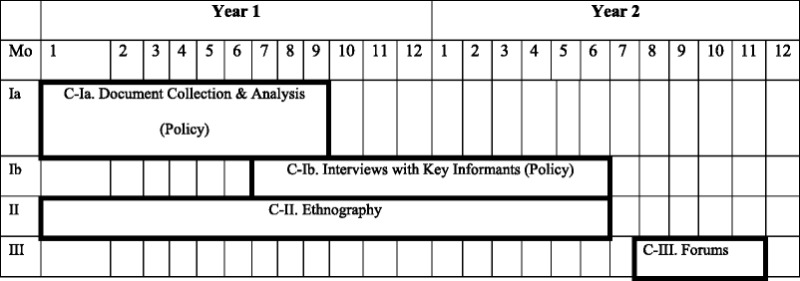
Timeline of components

### Component-I: ethical tensions in the development of recovery policy

First, we will first collect and analyze policy documents with the aim of examining ethical tensions that may exist in current policy. We will then use the discourses identified in the documents to prompt reflection about, and elicit key experiences on, ethical tensions that emerged during the creation of policy during narrative interviews with policy-makers (e.g., any individual who was involved in and contributed to the development of Canada’s Mental Health Strategy), with the aim of exploring any ethical tensions within their discursive practices. Our aim is to answer the key question for C-I: “What were the ethical tensions experienced by decision-makers during the formation of policy initiatives, and how were they resolved or not?”

### **Recruitment and accrual**

An initial list of participants will be generated using *purposive sampling* to ensure that we recruit members of MHCC, government employees, individuals who were asked to give testimony during the process, and individuals who participated in the public consultations during the policy-making process. We will then use *snowball sampling* whereby participants can suggest other participants. Recruitment will continue until the team decides that there is enough diversity and representation from the participant stories. We will aim to recruit at least 30 individuals (e.g., 10 government officials, 10 members of the MHCC, 10 public stakeholders, such as individuals living with mental illness or family members).

### **Data collection**

We will collect the following data:

*Policy documents*. We will collect documents (a) produced by or submitted to the MHCC, such as public speeches given by members of the Commission, reports from the different advisory committees, and documents by civil society organizations, professional groups, and other stakeholders [www.mentalhealthcommission.ca], and (b) generated during federal government and intergovernmental organization proceedings [www.openparliament.ca].

*Interviews*. We will ask participants to share retrospective stories about significant experiences and events during the process of policy development that posed dilemmas or challenged their reasoning about the “best good.” Significant or memorable experiences are often those that stand out from ordinary experience and are marked by the type of trouble that is central to stories [44, 45]. Questions will focus on eliciting stories: for example, participants will be asked, “Can you tell us about a memorable or significant event” [46, 47] that changed your understanding of recovery or posed a dilemma for you. The interviews will be recorded, transcribed verbatim, and de-identified.

### **Data analysis**

We will use both thematic and narrative analysis to examine the tensions between ethical values that may exist in policy documents and the ethical tensions that were experienced by policy-makers during the policy-making process.

*Policy documents*. We will conduct a thematic analysis to identify discourses (i.e., ideas, concepts, categories) in the documents using open coding to isolate and label pieces of text, such as the following: (a) common positions/statements about what mental health policy should foster, (b) who is asserting these positions/statements, (c) evidence used to support these positions/statements (e.g., experiential or scientific), and (d) text that situates these common positions/statements in a socio-historical context. We will then identify divergent discourses to locate ethical tensions as they pertain to different actors in the mental health system (e.g., government, healthcare professionals, public stakeholders).

*Interviews*. We will conduct a narrative analysis using the narrative-phenomenological framework to identify guiding metaphors or cultural genres around areas of conflict or issues that seemed to require some justification on the part of the interviewees. This analysis will provide important insights into the ethical tensions experienced by individuals as they made particular decisions and what (e.g., social norms, stories from individuals living with mental illness, scientific evidence) influenced these decisions. This analysis will also incorporate the findings from the thematic analysis in order to identify shared cultural understandings or principles (e.g., human dignity) pertaining to mental health policy. We note that these retrospective stories will be shaped by who conducts the interview, the location, and time relative to past and ongoing issues in their current work contexts, current policy debates, public events, or media. Hence, we will keep an audit trail on these details and interviewees will take reflective field notes.

Emerging results from C-I will be used during C-II to examine how different ethical tensions experienced during the development of policy map onto tensions that occur during its implementation.

### Component II: scenarios of ethical tensions with the implementation of recovery-oriented practice guidelines

Our ethnographic methodology draws from narrative and phenomenological traditions in anthropology [48]. Our intent, however, is not to describe either the culture of biomedicine or the institution as a set of values, attitudes, or beliefs. Rather, our stance is that although *cultural practices* are patterns of socially structured actions (habitus) [40, 49], we can use ethnographic strategies to “read” and thus provide a *thick description* of the meaning of actions and intentions in particular contexts [50]. Narrative phenomenology is ideal for understanding the *experiences* of particular persons, particularly during times of uncertainty or irruption. Thus, we chose a narrative and phenomenological framework to focus attention on the interactive dynamics and structural relationships in clinical practice [30] and how persons reason about the best good when the ends may be incommensurable [15, 29, 31]. Our key research question for C-II is as follows: What ethical tensions are emerging for mental health practitioners in the implementation of recovery-oriented standards and how are they being resolved (or not)? By focusing on practitioners’ experiences and ethical reasoning about recovery-oriented practice guidelines, our aim is to generate case-based scenarios, which we will feed-forward to the participatory forums in C-III.

### **Recruitment and accrual**

We will recruit mental health practitioners (e.g., nurses, psychiatrists, psychologists, social workers, occupational therapists, floor staff) from in-patient, out-patient, and emergency room units within a hospital psychiatry department that is located in one of the most culturally diverse areas of an urban city. This department has over 715 admissions, including emergency and high-risk care, as well as 80,500 ambulatory visits each year. We will use a combination of purposive and snowball sampling to identify individuals who are experiencing ethical tensions implementing recovery-oriented practice guidelines.

### **Data collection**

We will use the following three ethnographic methods:

*Participant observation*. Participant observation allows us to enter into the everyday routines of practitioners. Thus, we are better situated to experience and understand when there are disruptions or changes to habitual actions, such as when ethical tensions emerge or moral breakdowns occur [14]. The trained observers will take electronic *field notes of events* (e.g., team meetings, grand rounds), which will include details on contextual factors (e.g., material, institutional) and scene (e.g., verbal and nonverbal actions/interactions).

*Collective narratives*. Focus groups encourage members who have traditionally been marginalized to speak up, adding to the “multivocality of participants’ attitudes, experiences and beliefs” ([51]:836). We will use the emergent findings from C-I (i.e., ethical tensions identified in the policy documents and interviews with policy-makers) to elicit stories [46, 47] in order to access rich first-hand accounts of practitioners’ experiences during the shift from traditional to recovery-oriented care.

*Interviews*. We will conduct interviews with administrators, practitioners and support staff in order to understand how they experienced and reasoned about ethical tensions that arose during significant events that we observed in daily clinical practice or that emerged during focus groups. We will audiotape and de-identify transcripts for both the collective narratives and the interviews.

### **Data analysis**

Our analytic strategy is to iteratively move back and forth between the narrative-phenomenological analytic framework, data, and new theoretical resources which preliminary analyses indicate may be relevant to our aim, in addition to our overall reflection team meetings strategies for rigour as described below:

*Analysis of participant observations (field notes)*. Following standard methods of ethnographic research, participant observers will reflect on their field notes to pull out details and document patterns of ideas [52]. Other team members will probe for missing details to verify the links between personal values, ethical principles, and discursive practices and deepen description of the contextual factors as a *scene*. Scenes, like *genres*, are structural in nature in that scenes contain and guide actions [28]. Individual actions can also change the scene [53, 66]. Thus, we can analyze actions and interactions for if and how they change the traditional scene of practice towards one aligned with a recovery model. Thus, the analysis of participant observations is particularly important as it provides a window into what persons actually do everyday to supplement the stories they tell about their experiences in the interviews and collective narratives.

*Analysis of collective narratives and narrative interviews*. We will identify experience-near events, by marking shifts in tense or tone, direct quotations, and animated expression [46]. We will then analyze the narratives for biomedical ethical principles (e.g., beneficence), principles of recovery (e.g., empowerment, hope), what matters to particular persons, and discursive practices (e.g., genres of hope) [31]. In addition, we will monitor how narratives shift over the course of the study and/or if and when they shift between retrospective stories of past events and prospective stories about the future.

*Reflective team meetings*. Team members will generate thick descriptions around particular cases based on the above analyses, which they will then present to the team. We will develop working hypotheses, which we will refine across cases as they are developed. We will then collectively choose cases that are representative of key ethical tensions confronted by practitioners and their everyday ethical reasoning to generate case-based scenarios. In order to increase rigor [54], we will (a) keep detailed field notes, (b) maintain an audit trail (C-I-II), and (c) triangulate the different forms of data collected.

We also note, from our involvement with mixed methods collaborations, that there will be mixing at the level of epistemology, as team members bring in the expertise of their different disciplines, clinical practice, and other personal experiences [Zafran H, Tranulis C, Park MM. An illustration of team reflexivity using a double hermeneutics to make visible the epistemological mixing of data analysis. Qual Health Res. submitted]. In other words, we cannot bracket our own subjectivity but will take a reflexive approach or epistemic reflexivity [55, 56] to our engagements with participants and each other [57] in order to productively deepen our analysis [58, 59]. We will consistently review epistemological stances of team members, as this is also critical to maximizing the collaborative processes of interdisciplinary teams in health care research [59] and ask C-III participants to “consume the data and offer their feedback” ([60]:18).

Finally, we will use *validity criteria* [54] specific to mixed methods research. This includes summarizing and presenting the results and inferences for each of the research questions and components separately, with attendant critiques, prior to combining, comparing and/or contrasting the meaning of the overall results, and using emotions as validity checkpoints [60, 61]. The combination of all the results from CI-III (including negative, contradictory, or resistant situations, or stories) into a consistent, theoretically coherent meta-inference that is supported by expert consensus, and the literature to date is called *integrative efficacy* [54].

### Component-III: participatory forums to identify key ethical tensions and action steps

We follow the position that participatory research is not a methodology, but an approach to action for change [http://www.cihr-irsc.gc.ca/e/44954.html#a1], which can be compatible with narrative approaches: A first-person story “works as an action, if it can engender certain effects in the listener. […] But whether this occurs depends upon what sort of contract the listener is willing to make” ([62]:11). In C-III, our contract with the practitioners, policy-makers, persons with lived experience, and family members in the participatory forums will shift our interpretive paradigm towards a transformative one. We will use the narrative-phenomenological framework’s focus on understanding *events* from multiple perspectives to support “inclusion and communication in respectful ways” and “to inform the development of interventions that will be viewed as culturally responsive by the members of the communities we serve” ([63]:217), [64]. Our key research question for this component is as follows: What are the key ethical tensions as identified by decision-makers and mental health practitioners, and what do they establish as the action steps needed to close the gap between policy and practice?

### **Recruitment**

We will use purposive and snowball sampling, beginning with individuals who participated in C-I (policy-makers) and C-II (practitioners), who can suggest other stakeholders for recruitment (e.g., administrators, government officials, persons with mental illness, family members). Our recruitment efforts will be supported by and include team members who are leaders in the department’s Risk Management and Continuous Quality Improvement committee in both in-patient and out-patient units. We will also recruit from the Development Team of Accreditation Canada, which supports the larger Partnership in Health Systems Improvement grant in which this Catalyst Grant in Ethics is situated.

### **Participatory approach**

We will collectively discuss the policy findings from C-I and case-based scenarios from C-II with the recruited policy-makers and stakeholders from the Department of Psychiatry in the order as written below.

*Pre-forum data collection and analysis*. Following consent, we will provide passwords for participants to access de-identified C-I findings and C-II case-based scenarios on a password-protected website. We will invite individuals to identify critical points for reflection. Responses will be de-identified. The research team will then synthesize the list into themes using an open coding technique as in C-I and post them on the protected website.

*Forums*. We will use the themes to structure open forums, with the aim of cultivating a dialog on the ethical tensions pertaining to Canada’s Mental Health Strategy. Our objectives will be to collectively identify and prioritize key concerns and generate action steps to resolve them. Engaging stakeholders from the multiple perspectives of policy, clinic, and personal experience can challenge participatory processes [65]. We have found that having time to tell stories and to reflect upon them individually supports engaged dialog [20]. Thus, the logistical sequencing and feeding-forward of findings from C-I (ethical tensions in policy and reasoning of policy-makers) and C-II (case-based scenarios of ethical tensions and reasoning) to C-III is designed to cultivate both critique and reflection prior to the participatory forums. We will host two forums in order to accommodate the schedules of a variety of stakeholders.

*Dissemination*. Participatory processes are considered integrated knowledge translation to the extent that the key stakeholders involved are also the end users of the knowledge generated. Accreditation Canada and the MHCC have committed to utilizing outcomes. We will post the forum outcomes in a dedicated section on the PHSI website currently under construction with an open blog site for continued comments. Finally, we will integrate the findings of this Catalyst into the comprehensive report for the PHSI project, in which we are evaluating the process and outcomes of using a mixed methodological toolkit to tailor recovery-oriented care to particular contexts.

## Trial status

We are in recruitment and initial data collection for Components I-II at the time of this submission.

## Discussion

Trust and interpersonal relationships built over time with participants is critical to the success of a *relational ethics* approach and to ensuring optimal data for a first-person neo-Aristotelian virtue ethics analytic framework. As noted above, we developed this protocol in direct response to our partners of a CIHR and RxD Health Research Foundation PHSI grant, which is led by Park [10] and on which Lencucha is a Collaborator. Our Catalyst in Ethics team includes key clinicians as co-applicants and collaborators who hold key leadership positions in units, which have experienced significant ethical tensions related to the changes in policy. Thus, this protocol supports the systematic examination of the ethics of transforming mental health practice in sites where relationships have already been established. Relational perspectives also depend upon the inclusion of multiple perspectives, and our grant team includes clinicians with experience in policy and with policy-makers and persons with lived experience of mental illness with certification in peer support.

## Abbreviations

C-I: Component One
C-II: Component Two
C-III: Component Three
KT: Knowledge Translation
MHCC: Mental Health Commission of Canada
PHSI: Partnerships for Health System Improvement
